# Zinc as a Biomarker of Nutritional Status and Clinical Burden in Recessive Dystrophic Epidermolysis Bullosa: Implications for Preventive Monitoring

**DOI:** 10.3390/nu18020232

**Published:** 2026-01-12

**Authors:** Lucía Quintana-Castanedo, Rocío Maseda, Silvia Sánchez-Ramón, Nora Butta, Marta Molero-Luis, María G. Crespo, Antonio Buño, Sara Herráiz-Gil, Carlos León, Alberto Varas, Lidia M. Fernández-Sevilla, Pilar Zuluaga, Raúl de Lucas, Marcela del Río, Ángeles Vicente, María J. Escámez, Rosa Sacedón

**Affiliations:** 1 Department of Dermatology, Hospital La Paz, 28046 Madrid, Spain; luciaquintana.e@gmail.com (L.Q.-C.); rociomaseda@gmail.com (R.M.); rauldelucas@gmail.com (R.d.L.); 2 Department of Dermatology, Marqués de Valdecilla University Hospital, 39008 Santander, Spain; 3 Department of Immunology, Hospital Clínico San Carlos, 28040 Madrid, Spain; ssramon@salud.madrid.org; 4 Immunology Group, Health Research Institute of the Hospital Clínico San Carlos (IdISSC), 28040 Madrid, Spain; 5 Department of Hematology and Hemotherapy, Hospital La Paz, 28046 Madrid, Spain; nora.butta@salud.madrid.org; 6 Coagulopathies and Alterations in Haemostasis Group, IdiPAZ Health Research Institute, Hospital La Paz, 28046 Madrid, Spain; 7 Department of Laboratory Medicine, Hospital La Paz, 28046 Madrid, Spain; marta.molero@salud.madrid.org (M.M.-L.); mariagema.crespo@salud.madrid.org (M.G.C.); antonio.buno@salud.madrid.org (A.B.); 8 Neonatology Group, Hospital La Paz Institute for Health Research (IdiPAZ), La Paz University Hospital, 28046 Madrid, Spain; 9 Departamento de Bioingeniería, Universidad Carlos III de Madrid, 28911 Leganés, Spain; sherraiz@ing.uc3m.es (S.H.-G.); cleon@ing.uc3m.es (C.L.); mrnechae@ing.uc3m.es (M.d.R.); 10 Centro de Investigaciones Energéticas, Medioambientales y Tecnológicas (CIEMAT), 28040 Madrid, Spain; 11 Centro de Investigación Biomédica en Red de Enfermedades Raras (CIBERER)-ISCIII, 28029 Madrid, Spain; 12 Instituto de Investigación Sanitaria Fundación Jiménez Díaz (IIS-FJD, UAM), 28040 Madrid, Spain; 13 Department of Cell Biology and Histology, Faculty of Medicine, Universidad Complutense de Madrid (UCM), 28040 Madrid, Spain; avaras@ucm.es (A.V.); avicente@ucm.es (Á.V.); 14 Department of Basic Health Sciences, Faculty of Health Sciences, University Rey Juan Carlos, 28922 Alcorcón, Spain; lidia.martinez@urjc.es; 15 Department of Statistics and Operations Research, Faculty of Medicine, Universidad Complutense de Madrid (UCM), 28040 Madrid, Spain; pilarzul@med.ucm.es; 16 Stem Cells, Immunity and Cancer Group, Health Research Institute Hospital 12 de Octubre (I+12), 28041 Madrid, Spain

**Keywords:** recessive dystrophic epidermolysis bullosa, zinc deficiency, serum zinc levels, inflammation, albumin, anemia, skin fragility

## Abstract

**Background/Objectives**: Recessive dystrophic epidermolysis bullosa (RDEB) is a severe congenital genodermatosis characterized by skin and mucosa fragility, chronic inflammation, recurrent infections and high nutritional demands due to increased metabolism and epithelial barrier-related losses, placing patients at risk of zinc deficiency. We aimed to investigate the clinical relevance and biochemical determinants of zinc deficiency as a potentially modifiable contributor to disease burden in RDEB. **Methods**: In this cross-sectional study (*n* = 84), serum zinc levels were analyzed in association with sex, age, disease severity, percentage of body surface area (BSA) affected, inflammatory markers, infection burden, and common clinical complications including anemia and growth impairment. **Results**: Zinc deficiency, defined as levels below 670 µg/L, was identified in 35% of patients and became more frequent after age 5 and during adulthood, particularly among those with more severe disease. Deficiency was strongly associated with anemia, inflammation, infection burden, growth impairment, and extensive skin involvement. A revised cutoff of 780 µg/L is proposed, showing improved diagnostic performance for identifying patients at risk of systemic complications, and offering a more suitable threshold for starting preventive supplementation. Multivariate logistic modeling confirmed that low serum zinc independently predicted anemia risk, alongside transferrin saturation and C- reactive protein levels. Serum albumin was identified as the strongest determinant of zinc levels, partially mediating the effects of inflammation and skin involvement. **Conclusions**: These findings identify serum zinc as a clinically relevant marker of nutritional status and complication burden in RDEB. While no causal or therapeutic effects can be inferred from this cross-sectional study, the strong and biologically plausible associations observed suggest a rationale for systematic monitoring and correction of zinc deficiency as part of comprehensive supportive care, and warrant prospective studies to assess clinical benefit.

## 1. Introduction

Patients with recessive dystrophic epidermolysis bullosa (RDEB) suffer from recurrent, painful wounds from birth as a direct consequence of the hereditary loss of function of type VII collagen anchoring fibrils [1]. Beyond this cutaneous fragility, this genetic disorder evolves into a chronic multisystemic condition in which persistent inflammation [2,3] and infection [4,5] not only exacerbate epithelial damage but also give rise to numerous complications including early-onset malnutrition that further fuels disease progression [6,7,8,9,10,11]. Advanced therapies are emerging as potential treatments for RDEB [6,12,13]. Although recently approved local gene therapies represent a major milestone, their long-term efficacy, systemic benefits, and accessibility remain uncertain [14,15,16]. Crucially, their therapeutic impact may be limited by systemic factors associated with disease severity. This underscores the need for safe, systemic approaches that address modifiable contributors to disease burden before severe or irreversible stages develop. Inflammation and malnutrition are key candidates, as both are well known to exacerbate disease manifestations and compromise tissue homeostasis and repair. Ideally, such strategies—simple, affordable, and applicable across disease stages and severities—could complement both current and future therapies, ultimately improving prognosis and quality of life.

Zinc is an essential trace element involved in tissue repair, hematopoiesis, growth, immune function, endocrine homeostasis, and epithelial barrier maintenance [17,18,19]. Its ubiquity and multifunctionality are evidenced by its role as a cofactor for numerous enzymes. These include key proteins with fundamental cellular functions such as protein kinase C, caspase 6 and 8, superoxide dismutase, matrix metalloproteinases, and DNA methyltransferases [20]. Additionally, zinc supports the activity of more than 1000 transcription factors, including zinc finger proteins like GATA, Krüppel-like factor family members, or Specificity Protein 1 like proteins [20]. Zinc deficiency can therefore lead to a broad range of systemic effects, including impaired wound healing, growth delay, sarcopenia, anemia, susceptibility to infections, anorexia, inflammation, and psychological symptoms [17,18,21], all of which are common in RDEB patients with multifactorial malnutrition [7,8,22,23,24].

The skin contains approximately 5% of the body’s total zinc [25]. In RDEB, zinc requirements are heightened due to continuous losses from recurrent and often extensive skin wounds, alongside an increased need to support tissue repair and immune defense. Systemic inflammation further contributes to functional zinc deficiency by promoting hepatic sequestration, reducing intestinal absorption, and increasing excretion in response to cytokines such as IL6 [19]. At the same time, reduced oral intake due to odynophagia, mucosal damage, and feeding difficulties limits dietary zinc supply in many patients. Unlike iron, zinc lacks substantial tissue storage [26]; thus, even transient imbalances may impair essential biological functions. In chronic settings such as RDEB, a persistent inflammatory and catabolic state, compounded by inadequate intake, places patients at particularly high risk for zinc deficiency, which may, in turn, exacerbate disease progression [7,22,27,28].

Despite zinc’s recognized importance, its deficiency and clinical impact in RDEB are poorly defined, and no evidence-based, standardized supplementation protocols are currently available [7,8]. This study evaluates zinc levels in a cross-sectional RDEB cohort and investigates their association with disease severity, skin complications, growth delay, anemia, inflammatory and infection burden, and psychological manifestations. By identifying patients at risk for zinc deficiency and elucidating its systemic effects, we aim to underscore the need for preventive and tailored interventions to improve clinical management and quality of life in patients with RDEB.

## 2. Materials and Methods

### 2.1. Study Design, Patients, and Data Collection

This cross-sectional, observational, and non-interventional study has been described in detail elsewhere [3,29]. Briefly, from May to December 2021, all patients with a confirmed diagnosis of RDEB registered at the Spanish Reference Unit for EB (HULP) were considered for inclusion (*n* = 93). Clinical evaluations and sample collection were carried out during routine follow-up visits. Disease severity was assessed by a single dermatologist using the Epidermolysis Bullosa Disease Activity and Scarring Index (EBDASI), with established cut-offs applied [30] and disease-associated complications documented. Demographic information (Table S1), the percentage of body surface area (BSA) affected, the use of oral zinc supplementation (OZS), and the presence of active wound infection at the time of assessment were recorded. Patients were also asked about psychological symptoms, including depression, anxiety, and low mood. In addition, information was collected regarding previous culture-confirmed skin infections (history of cutaneous infection) and a history of hospitalizations due to severe infections (non-responsive to oral antibiotics or sepsis).

During the visit, anthropometric parameters were determined, and Z-scores for weight and height by age were calculated using contemporary Spanish reference charts [31] through the nutritional application provided by the Sociedad Española de Gastroenterología, Hepatología y Nutrición Pediátrica (Spanish Society of Pediatric Gastroenterology, Hepatology and Nutrition; SEGHNP; https://www.seghnp.org/nutricional/ accessed on 6 Jun 2022)). Peripheral blood was drawn only when the patient’s clinical condition allowed.

Routine biochemical and hematological parameters were analyzed in the Central Clinical Laboratory at HULP, applying standard reference ranges (Table S1). Interleukin-6 (IL6) levels were quantified using a commercial kit (Merck KGaA (Sigma-Aldrich, Madrid, Spain)) at the Hemostasis Laboratory according to established protocols as previously described [3], and an age- and sex-matched group of healthy controls served as the reference cohort. In this case, patient and control samples were processed in parallel to reduce variability. IL6 values exceeding the 95th percentile of the healthy control cohort distribution were considered abnormally high [3].

### 2.2. Zinc Quantification

Blood samples were obtained after overnight fasting, between 08:00 and 10:00 a.m., to minimize circadian variation in serum zinc and to avoid the effect of recent food intake. Samples with marked hemolysis (hemolysis index > 200 mg/dL hemoglobin) were excluded, as the release of intracellular zinc from erythrocytes can lead to overestimation of serum concentrations.

Serum zinc was measured by flame atomic absorption spectrometry using a Perkin Elmer AAnalyst 200 spectrometer (PerkinElmer, Waltham, MA, USA) with a deuterium background corrector and a zinc-specific hollow cathode lamp. Calibration curves were prepared weekly from certified 1000 ppm standards and accepted when the correlation coefficient was >0.99 and the variation compared with previous calibrations was <20%. Internal controls (Bio-Rad Liquid Assayed Multiqual (Alcobendas, Spain), levels 1–2) were included in each run, and external quality was monitored through participation in the SEQC trace elements program. All samples were analyzed in duplicate; results with a relative standard deviation (RSD) >3% were repeated, and dilutions were performed when concentrations exceeded the linear range. The method is accredited under the UNE-EN ISO 15189:2023 standard (Medical laboratories–Requirements for quality and competence. International Organization for Standardization (ISO): Geneva, Switzerland, 2023), within the scope of the laboratory’s accreditation.

### 2.3. Statistical Analysis and Visualization

Statistical analyses and data visualization were performed using Microsoft Excel 365, GraphPad Prism 8.0.2, SPSS v25.0, and Python 3.11.4 via the Data Analyst environment (OpenAI). Full details on all statistical procedures, software versions, and AI-assisted modeling and visualization are available in the Supplementary Methods.

## 3. Results

### 3.1. Zinc Status Is Related to Age Group and Disease Severity in RDEB

**Figure 1:**
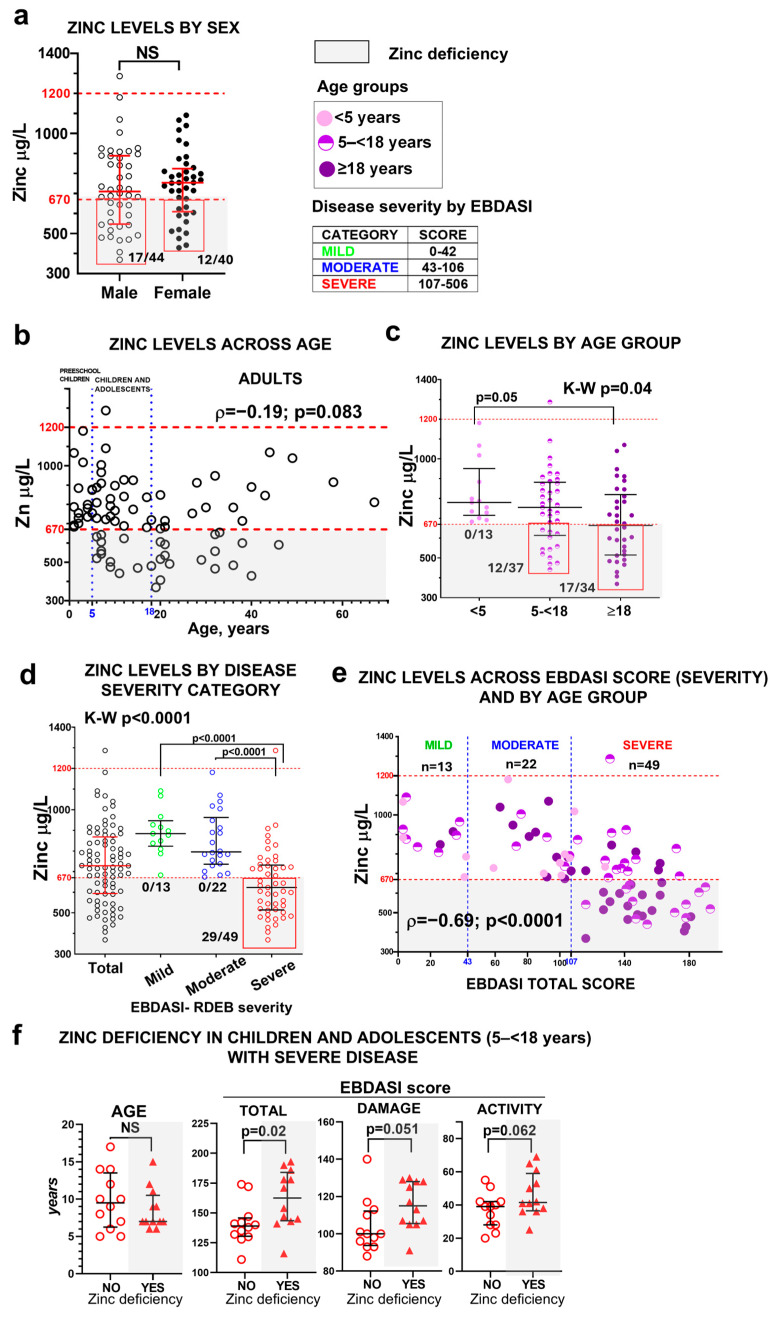
Zinc status in the RDEB cohort. Serum zinc levels are shown according to sex (**a**); across age as a continuous variable (**b**); by age group (preschool children <5 years, children and adolescents 5–<18 years, and adults ≥18 years) (**c**); by EBDASI severity categories (mild <43 points, moderate 43–106 points, and severe ≥107 points) (**d**). Panel (**e**) shows the relationship between serum zinc levels and the EBDASI score as a continuous variable, also stratified by age group. Statistical analyses include an unpaired t-test for panel (**a**), the Kruskal–Wallis test followed by Dunn’s multiple comparison test for panels (**c**,**d**), and Spearman’s rank-order correlation coefficient (ρ) for panels (**b**,**e**). NS indicates non-significant results (*p* > 0.05). Error bars represent the median and interquartile range, and dashed lines denote the normal zinc reference range (red) and EBDASI severity thresholds (blue). Panel (**f**) shows the comparison of age (Mann–Whitney test) and EBDASI severity scores (unpaired *t*-test) in children and adolescents with severe disease, stratified by zinc status (with or without zinc deficiency, *n* = 12 per group) with error bars representing the median and interquartile range. Gray shading indicates zinc deficiency.

Zinc deficiency was identified in 29 of the 84 patients (34.5%). No significant differences were observed between sexes in either zinc levels or deficiency prevalence (Figure 1a). One patient with severe RDEB and no zinc supplementation showed zinc levels above the normal range, though no complications were associated with this finding. Zinc levels did not correlate with age as a continuous variable (Figure 1b) but differed by age group (Figure 1c). The risk of zinc deficiency increased with age (χ^2^ test, *p* = 0.003), affecting no preschool children (1–<5 years), 32% of children/adolescents (5–<18), and 47% of adults (Figure 1c).

Zinc levels were significantly associated with disease severity, as defined by EBDASI score. All patients with zinc deficiency fell within the severe EBDASI category (≥107 points; Figure 1d, Table S2), and lower zinc concentrations correlated with higher EBDASI total scores (Figure 1e).

Given the developmental vulnerability of the children–adolescent group, when the progression of RDEB typically accelerates and the risk of complications and mortality increases, we focused on patients in this age range with severe disease. Although the age distribution was similar between zinc-deficient and non-deficient patients within this subgroup, those with zinc deficiency exhibited significantly higher EBDASI total scores as well as both activity and damage subscores (Figure 1f), indicating that zinc deficiency is associated with greater disease activity and cumulative tissue damage. This stage carries a higher physiological demand for zinc due to growth and tissue turnover. Zinc scarcity may therefore contribute to worsening disease burden and influence the natural course of RDEB.

OZS data are provided in the Supplementary Material, Figure S1. Overall, OZS often failed to match patients’ clinical needs, with many zinc-deficient patients not taking supplementation, and some supplemented individuals remaining deficient.

### 3.2. Zinc Deficiency and Its Clinical Consequences

#### 3.2.1. Zinc Deficiency as a Risk Factor for Growth Impairment in RDEB Patients

**Figure 2:**
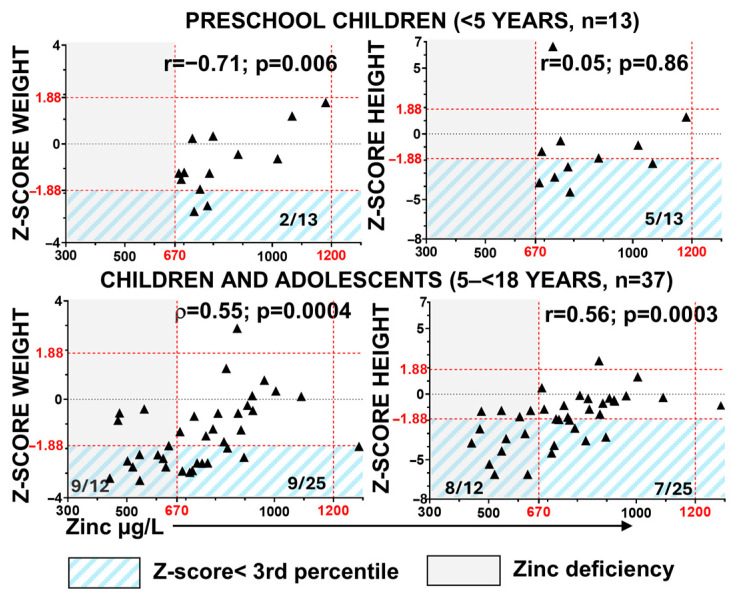
Association of zinc deficiency with growth impairment in RDEB. Correlation between serum zinc levels and anthropometric parameters was assessed using Z-scores for weight and height by age, calculated from contemporary Spanish reference charts [31]. Statistical analyses include Pearson’s correlation coefficient (r) and Spearman’s rank-order correlation coefficient (ρ), with corresponding *p*-values. Red dashed lines indicate the normal zinc reference range (vertical) and the 3rd and 97th percentile thresholds for Z-scores (horizontal). Gray shading indicates zinc deficiency. Blue diagonal shading highlights patients with Z-scores below the 3rd percentile.

We assessed the correlation between zinc levels and Z-scores by age for weight (ponderal growth) and height (linear growth) (Figure 2). In preschool children, 15% (2/13) and 38% (5/13) had weight and height values below the 3rd percentile, respectively. However, only weight Z-scores exhibited a significant correlation with zinc levels in this group. In children and adolescents, the percentages of growth impairment increased in parallel with the rising prevalence of zinc deficiency (Figure 2), and a moderate positive correlation was observed between zinc levels and both height and weight Z-scores. Impaired ponderal growth occurred in 75% of zinc-deficient patients versus 36% of patients with normal blood zinc levels (χ^2^, *p* = 0.04), and stunted linear growth was observed in 67% versus 28%, respectively (χ^2^, *p* = 0.04). These findings suggest that zinc deficiency is associated with impaired growth in paediatric patients with RDEB.

#### 3.2.2. Zinc Deficiency Is Strongly Associated with Common Complications in RDEB

**Figure 3:**
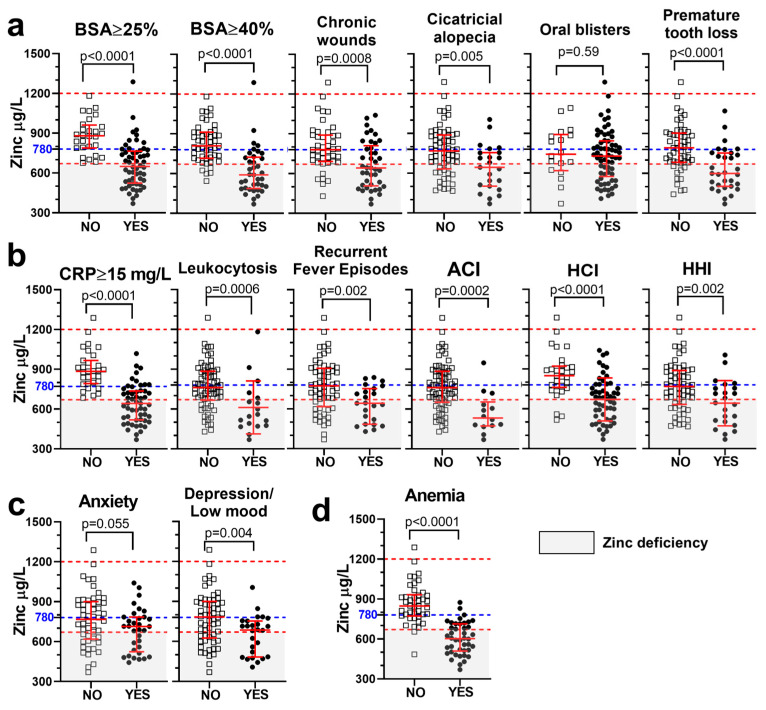
Lower serum zinc levels in patients presenting with the most frequent complications in RDEB. Zinc levels, analyzed as a continuous variable (µg/L), are compared between patients with (“Yes”, open squares) or without (“No”, solid circles) specific clinical outcomes: cutaneous and oral complications (**a**), inflammatory and infectious burden (**b**), psychological symptoms (**c**), and anemia (**d**). Statistical analysis (*p*-value) was performed using an unpaired *t* test. Dashed lines indicate the normal zinc range (red) and the proposed threshold for preventive supplementation (780 µg/L; blue). %BSA = percentage of body surface area affected; CRP = C-reactive protein; ACI = active cutaneous infection; HCI = history of cutaneous infection; HHI = history of hospitalization due to serious infection (nonresponsive to oral antibiotics or sepsis). Gray shading indicates zinc deficiency.

As shown in Figure 3, lower serum zinc levels, analyzed as a continuous variable, were found in patients with the most typical complications in RDEB. These included outcomes affecting the skin and oral mucosa (Figure 3a), complications related to inflammatory and infection burden (Figure 3b), as well as alterations in the psychological domain (Figure 3c) and anemia (Figure 3d).

Statistically significant associations with zinc deficiency, analyzed as a binary variable (<670 µg/L cutoff), were strongest in the domains of cutaneous and mucosal involvement, inflammatory and infectious burden, and anemia, indicating that these complications were more prevalent among patients with zinc deficiency. In contrast, psychological complications did not show significant associations in the binary analysis (Table 1). Notably, higher proportions of patients with extensive skin involvement (BSA ≥ 25% or ≥40%) and chronic wounds were found among those with zinc deficiency (Table 1).

Permanent complications such as cicatricial alopecia and premature tooth loss were also more prevalent in the zinc-deficient group, supporting the long-term consequences of suboptimal zinc status. In contrast, oral blisters, despite their frequency in RDEB, were not significantly associated with zinc levels (Figure 3a, Table 1).

Supporting the known role of zinc in immune function, lower zinc levels were associated with an increased proportion of patients with moderate-to-high levels of CRP and an increased infection burden, evidenced by higher rates of leukocytosis, active cutaneous infection, history of cutaneous infection, and history of hospitalization due to severe infection (Figure 3b, Table 1).

The strongest associations with zinc deficiency were observed for anemia (odds ratio (OR) = 36, *p* < 0.0001), CRP ≥ 15 mg/L (OR = ∞, *p* < 0.0001), extensive skin involvement (BSA ≥ 25%: OR = ∞; BSA ≥ 40%: OR = 15; both *p* < 0.0001), and chronic wounds (OR = 10, *p* < 0.0001). The highest Youden’s J indices were observed for anemia (J = 0.60), CRP ≥ 15 (J = 0.55), and BSA ≥ 40% (J = 0.54), indicating moderate diagnostic utility (Table 1).

### 3.3. Zinc Threshold for Preventive Risk Stratification in RDEB

Despite these strong associations, the sensitivity of the current zinc deficiency cutoff according to the laboratory’s reference interval (670 µg/L) remains below 70% for most outcomes (Table 1), limiting its effectiveness as a clinically meaningful preventive supplementation threshold and increasing the risk that vulnerable RDEB patients may go unrecognized. This pattern is consistent with zinc’s classification as a Type 2 nutrient, in which clinical manifestations such as impaired growth, immune dysfunction, and delayed wound healing often precede measurable declines in circulating levels [32].

To improve risk detection and guide preventive intervention, we calculated zinc thresholds corresponding to 80% sensitivity for complications that not only showed strong statistical associations with zinc status but also represent early drivers of morbidity in RDEB, particularly extensive skin involvement, systemic inflammation/infection-related outcomes, and anemia. Analyses were performed in the overall cohort and stratified by two age groups (children and adults) to capture possible differences in physiological requirements and disease presentation.

Across complications, the zinc levels required to achieve the sensitivity target ranged from 728 to 790 µg/L, with some variation by age group (Table S4). Among these, a fixed threshold of 780 µg/L emerged as a consistent and clinically practical option. It provided high sensitivity (≥80%) and moderate-to-high specificity for the key systemic complications mentioned above. Additionally, negative predictive values were consistently high at this threshold, supporting its use as a reliable screening tool to rule out clinical risk (Table S5).

Altogether, a threshold of 780 µg/L shows strong diagnostic performance for identifying patients at risk of systemic complications in RDEB, while prospective studies are required to determine its role in guiding preventive zinc supplementation.

### 3.4. Zinc Levels as a Risk Predictor of Anemia in RDEB

**Figure 4:**
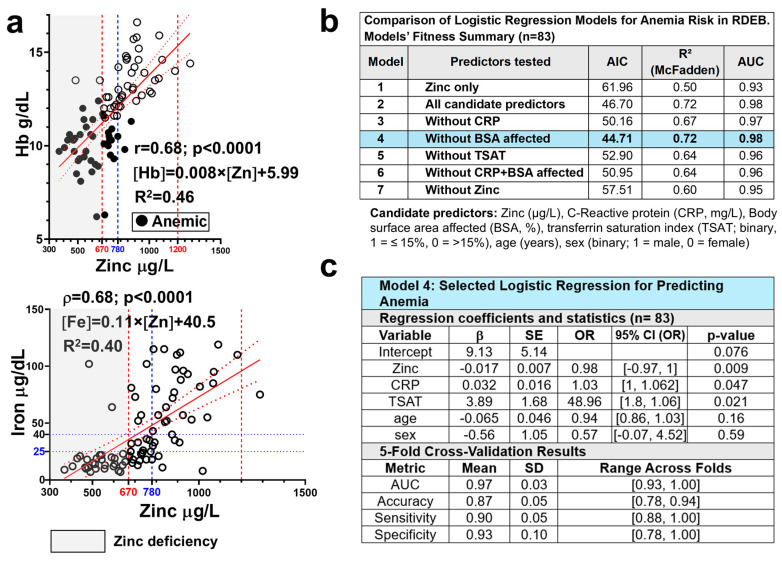
Zinc levels as a predictor of anemia risk in RDEB. (**a**) Correlation of serum zinc levels (µg/L) with hemoglobin (Hb, g/dL; *n* = 84) and serum iron (µg/dL; *n* = 83) in patients with RDEB. Spearman’s rank-order correlation coefficient (ρ) and corresponding *p*-values are shown; linear regression lines (solid red lines) with 95% confidence intervals (dotted red lines) are displayed. Vertical dashed lines indicate the normal zinc range (in red) and the proposed threshold for preventive supplementation (780 µg/L; blue) Gray shading indicates zinc deficiency. (**b**) Comparison of logistic regression models with different predictor sets and (**c**) summary of the final selected model (Model 4), including 5-fold cross-validation results. Blue shading highlights the selected model. CRP = C-reactive protein; BSA = Body surface area; TSAT = transferrin saturation; AIC = Akaike Information Criterion; R^2^ (McFadden) = pseudo-R^2^; AUC = area under the receiver operating characteristic curve. β = represent log-odds from the logistic regression model predicting anemia risk (1 = anemic, 0 = non-anemic); SE = Standard error of the β coefficient; OR = odds ratio (e^β^); *p*-value = statistical significance of each predictor; 95% CI (OR): confidence interval for OR estimates, reported as [lower, upper] SD = Standard deviation.

We previously reported that anemia is a prevalent and multifactorial complication in this cohort, affecting 50% of patients [29]. The main aetiopathogenic factors identified as contributing to anemia in RDEB include the extent of skin involvement, systemic inflammation, and both functional and true iron deficiency [29,33,34,35]. In addition to these factors, we examined whether zinc deficiency contributes to anemia risk in this cohort. Hemoglobin and zinc levels showed a strong correlation (Figure 4a), and patients with anemia exhibited significantly lower zinc levels compared to those without anemia (Figure 3d). Notably, 93% (27/29) of patients with zinc deficiency had anemia, representing a threefold higher prevalence compared with patients with zinc levels within the normal range (Table 1). Hypoferremia and zinc deficiency coexisted in many patients in this cohort, although hypoferremia was more common (Figure 4a). A univariate logistic regression model including serum zinc levels accounted for substantial variability in anemia risk (pseudo-R^2^ = 0.5) and showed a high discriminative capacity (area under the receiver operating characteristic curve (AUC) = 0.93), highlighting the strength of this association (Figure 4b).

To determine whether zinc levels remain an independent predictor of anemia risk when accounting for other relevant clinical variables, namely inflammation, extent of skin involvement, and iron bioavailability, multifactorial logistic regression models were constructed and compared. Models included zinc, CRP, %BSA affected, TSAT ≤ 15%, age, and sex, and were tested iteratively by removing predictors (Figure 4b, Table S6). Interaction terms (Zinc × CRP and Zinc × TSAT) were also evaluated and found to be non-significant (p = 0.83 and p = 0.91, respectively), suggesting that the effect of zinc was not dependent on inflammation or iron status. Accordingly, they were excluded from the tested models for clarity and parsimony in the model including all candidate predictors (Model 2, Table S6), zinc levels remained significantly associated with anemia risk (*p* = 0.012), and TSAT ≤ 15% strongly predicted anemia (*p* = 0.014), with patients above this threshold being approximately 49 times more likely to be non-anemic. In contrast, CRP showed only a marginal association (*p* = 0.058), and %BSA affected was not significant in this or any other model tested (Table S6). Excluding %BSA (Model 4) improved fit (lower Akaike information criterion, AIC) while preserving its performance (pseudo-R^2^ = 0.72; AUC = 0.98) (Figure 4b). Excluding zinc (Model 7) reversed the intercept, increased CRP’s coefficient, and worsened model performance (lower AUC and higher AIC), highlighting zinc’s relevant role in both prediction and model stability (Figure 4b).

Mediation analysis (Baron & Kenny method and Sobel test; Table S7) further supported that reduced zinc availability partially mediates the negative effects of inflammation and skin damage on erythropoiesis and anemia risk.

Model validation (5-fold cross-validation) confirmed robustness and generalizability, with cross-validated AUC of 0.97 and mean predictive accuracy exceeding 85% across folds. These findings establish blood zinc concentration as an independent and consistent predictor of anemia risk in RDEB, adding predictive value beyond inflammation and iron availability markers. Specifically, each 1 µg/L increase in zinc was associated with an approximately ≈ 2% higher likelihood of being non-anemic.

To illustrate the clinical relevance of this association, we estimated the predicted anemia risk in patients with similar inflammatory and iron-deficient profiles, varying only in their zinc levels. For a 20-year-old female with CRP = 15 mg/L and TSAT ≤ 15%—A high-risk profile in this cohort—the predicted probability of anemia was approximately 69% when serum zinc was 670 µg/L. In contrast, if zinc was 780 µg/L, the predicted risk dropped to 26%. This corresponds to a ~43% absolute risk reduction associated with higher zinc levels, underscoring the proposed independent protective effect of zinc and supporting the clinical relevance of the 780 µg/L threshold as a potential target for intervention.

### 3.5. Analysis of Factors Determining Blood Zinc Levels

**Figure 5:**
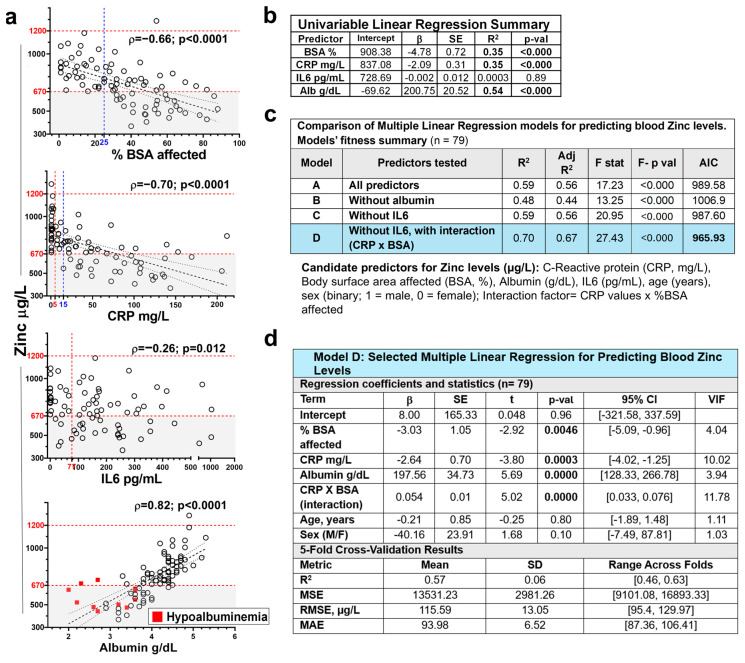
Factors influencing blood zinc levels in RDEB. (**a**) Scatterplots showing the association between blood zinc levels (µg/L) and potential predictors, including percentage of body surface area affected (BSA; %, *n* = 84), serum C-reactive protein (CRP; mg/L, *n* = 84), interleukin-6 (IL6; pg/mL, *n* = 79), and albumin (g/dL, *n* = 84), with fitted linear regression lines (dashed black) and 95% confidence intervals (dotted). Horizontal dashed lines indicate the normal zinc reference range. Gray shading indicates zinc deficiency. Dashed vertical red lines indicate the normal range for CRP (5 mg/L) and the 95th percentile of healthy controls used as the cut-off for IL-6 (77 pg/mL; [3]). (**b**) Statistical output of the corresponding univariable linear regression models predicting blood zinc levels. (**c**) Comparison of logistic regression models including different predictor sets (with or without albumin, IL6 and CRP × BSA interaction; *n* = 79). (**d**) Summary of the final selected model (Model D), including 5-fold cross-validation results. Blue shading highlights the selected model. β = unstandardized regression coefficient; SE = standard error of the regression coefficient β; *p*-val= *p*-value for testing β ≠ 0; R^2^ = coefficient of determination; Adj R^2^ = Adjusted R^2^; F stat = Fisher’s F-test result; F- *p* val = Fisher’s F-test *p* value; AIC = Akaike Information Criterion; t = T-statistic (β/SE); 95% CI = confidence interval; VIF = Variance Inflation Factors; MSE = mean squared error; RMSE = root mean squared error in the original units of zinc. MAE = mean absolute error; SD = standard deviation.

To identify clinical and biochemical predictors of zinc levels in RDEB, we explored variables selected based on their pathophysiological likelihood and observed correlation in the cohort (Figure 5a): the extent of skin damage (percentage BSA affected), inflammatory markers (CRP, IL6), and the main circulating zinc carrier and negative acute-phase protein (serum albumin). In univariate linear regression, zinc levels showed moderate negative associations with the percentage of BSA affected and CRP levels, and a strong positive association with serum albumin, while IL6 did not reach statistical significance (Figure 5b).

The comparative analysis of the performance of candidate predictors in multivariate models, including or not including albumin and IL6, and adjusted for age and sex, confirmed albumin as the strongest predictor of zinc levels, while discarding the independent relevance of IL6 (Figure 5c,d and Table S8). In addition, as suggested by exploratory data analysis, the inclusion of a CRP × BSA interaction term significantly improved the model ‘s fit (adjusted R^2^ = 0.70; ΔAIC = −21.7) (Figure 5c). Notably, the interaction coefficient was positive (β = 0.05), indicating that although both inflammation and skin involvement were independently associated with lower zinc levels, their combined effect became less than additive at extreme values.

The selected model (Model D; Figure 5d) generalizability along cohort subgroups, was assessed by 5-fold cross validation, achieving a mean R^2^ of 0.57 and root mean squared error (RMSE) of 115.59 µg/L across folds.

Finaly, mediation analysis (Baron & Kenny approach and Sobel test) confirmed that albumin significantly mediated the effects of CRP and BSA on zinc (Table S9), jointly explaining 72% of the albumin variance. These findings support a model in which skin damage and inflammation affect zinc levels primarily through their impact on serum albumin but may also exert a direct effect.

## 4. Discussion

This cross-sectional study characterizes zinc status in a representative cohort of patients with RDEB, a devastating congenital and chronic genodermatosis. Prior to this study, the clinical relevance of zinc in RDEB had largely been inferred from indirect evidence, extrapolated from its well-established roles in epithelial repair, immune function, and hematopoiesis, in the absence of direct, disease-specific investigations. Evidence from the analysis of this cohort demonstrates a clear association between zinc deficiency, disease severity, and a wide spectrum of complications, including chronic wounds, inflammation, infection burden, and growth impairment, many of which have a direct impact on quality of life and prognosis. Moreover, zinc levels emerged as the strongest predictor of anemia risk in RDEB patients.

### 4.1. Determinants of Zinc Status in RDEB

Zinc deficiency is common in chronic inflammatory and malnourished states, particularly in children, but it remains frequently underdiagnosed [36,37]. Zinc deficiency was observed in one-third of the total cohort and in two-thirds of patients with severe EBDASI scores, a proportion lower than previously reported in other cohorts with diverse demographic and clinical characteristics [27,28,38]. IL6 was only weakly associated with serum zinc levels, whereas CRP and extent of skin involvement (BSA affected) were identified as relevant and additive factors determining zinc deficiency. These associations were largely mediated by serum albumin, the main zinc carrier in circulation and the strongest independent predictor of its levels in RDEB. Albumin decline may precede zinc scarcity, as it reflects protein loss from wounds and inflammation-driven hepatic downregulation [28].

Zinc deficiency itself may further impair albumin synthesis [39,40], creating a vicious cycle that undermines systemic zinc transport and bioavailability. Consistently, albumin status could also influence the response to OZS. Because serum albumin reflects the overall protein–energy status, zinc supplementation should be integrated with comprehensive nutritional intervention, as has been previously suggested [8,41].

### 4.2. Relationship Between Zinc Deficiency and Skin Involvement in RDEB

Zinc’s role in wound healing is well established [42,43,44]. Topical formulations, such as zinc oxide, widely used in dermatology for exudative wounds and chronic ulcers, provide anti-inflammatory, antimicrobial, and re-epithelialization benefits even in normozincemic individuals [42,44,45]. Such mechanisms are particularly relevant in RDEB, where effective management of chronic wounds remains a central therapeutic challenge. However, although zinc-containing dressings are listed among optional products in EB resources, evidence for their use in RDEB is lacking and their clinical application has not been formally documented. Extensive application of zinc-containing dressings could increase systemic zinc absorption [46] and potentially result in toxicity [47]; their use should therefore remain localized and be carefully monitored.

Chronic wounds may be both a cause and a consequence of zinc deficiency. In our cohort, zinc deficiency was strongly associated with extensive skin damage: patients with wounds affecting ≥25% of their body surface had a markedly increased risk of zinc deficiency. Conversely, zinc-deficient individuals more frequently exhibited delayed healing and severe skin involvement (e.g., BSA ≥ 40%), suggesting a self-perpetuating loop of zinc loss and impaired repair.

### 4.3. Systemic Consequences of Zinc Deficiency: Growth Impairment, Immune Dysregulation, and Anemia in RDEB

In children with RDEB, we observed a correlation between serum zinc levels and weight- and height-for-age Z-scores from the age of five onward, an association previously reported for weight by Reimer et al. [28] This supports a link between zinc deficiency and growth retardation, although stunting typically begins earlier than the onset of measurable zinc depletion [48,49]. Given the multifactorial nature of growth impairment in RDEB, including inflammation and nutritional compromise, both intermingled with zinc status, it is difficult to establish a direct causal pathway. Nonetheless, zinc deficiency is known to impair the synthesis and signaling of thyroid hormones [50], androgens [51], and growth hormone [52,53], and zinc supplementation has been shown to improve growth and increase insulin-like growth factor 1 levels in pediatric populations [54,55,56].

Zinc is essential for immune function and inflammatory balance, a role that is particularly relevant in RDEB, where persistent inflammation and increased vulnerability to infections are major drivers of morbidity [19,21]. Zinc deficiency compromises the mechanisms that restrain NF-κB signaling, thereby enhancing proinflammatory cytokine production [57,58,59]. In RDEB, where damage-associated molecular patterns such as HMGB1 can activate NF-κB [60,61], zinc scarcity may further amplify these pathways, sustaining local and systemic inflammation. In parallel, zinc is essential for effective innate and adaptive immune responses, including neutrophil extracellular trap (NET) formation, thymic integrity, and B-cell differentiation [19]. Its deficiency impairs antimicrobial defense and increases susceptibility to recurrent infections and sepsis [19,21], major contributors to morbidity and mortality in RDEB [4,5,62]. Prophylactic zinc supplementation has been shown to reduce infection incidence in other settings, whereas its effectiveness appears limited once infection is established [63]. Whether similar benefits apply to RDEB requires prospective evaluation. In our cohort, zinc-deficient patients with severe disease were more likely to exhibit higher inflammatory markers and clinical signs of immune dysfunction, including leukocytosis, recurrent febrile episodes, active skin infections, and a history of serious infections. This strengthens the rationale for considering zinc status as a key modulator of inflammation and a determinant of immune competence in RDEB.

Anemia, a highly prevalent and multifactorial complication in RDEB [29,33,34,35], has been associated with zinc deficiency in different contexts [64,65,66,67]. Our study extends this evidence by demonstrating that zinc deficiency is also a significant contributor to anemia in RDEB. Zinc supports erythropoiesis through several mechanisms: it acts as a cofactor for enzymes involved in heme synthesis and oxidative stress protection and serves as a cofactor for transcription factors such as GATA-1 and FOG-1, which mediate erythropoietin (EPO) signalling and are indispensable for early erythroid commitment [68,69]. Zinc also influences the production and activity of insulin-like growth factor 1 and the growth hormone pathway, which synergize with EPO during erythropoiesis [54,69,70]. Consistent with these mechanisms, logistic regression analysis identified low serum zinc as an independent predictor of anemia risk in RDEB, with an effect size comparable to CRP and TSAT, even after adjustment for inflammation and iron status. This role is further supported by the interplay between zinc and iron metabolism, particularly relevant in RDEB, where coexisting deficiencies may exacerbate anemia and reduce response to standard treatments. Zinc repletion has been shown to improve iron mobilization and hemoglobin synthesis by upregulating the expression of key transporters such as divalent metal transporter 1 (DMT1) and ferroportin [69,71,72]. In chronic inflammatory conditions like chronic kidney disease, zinc supplementation enhances erythropoietic responses even in patients refractory to EPO therapy [67,73]. Accordingly, these findings support the biological plausibility of zinc deficiency as a relevant factor associated with anemia in RDEB, given its established roles in iron mobilization, inflammatory regulation, and erythropoiesis. Taken together, they provide a rationale for monitoring zinc status and correcting deficiency as part of anemia management, pending confirmation in interventional studies.

### 4.4. Rationale for Guiding Zinc Preventive Supplementation in RDEB

Our data show that zinc deficiency often emerges during childhood in patients with RDEB, a period marked by increased physiological demands due to growth and disease progression. Although zinc supplementation is widely recommended for patients with chronic wounds, inflammation, or growth impairment [22], specific criteria to initiate preventive therapy (such as thresholds for skin involvement, severity scores, CRP levels, or age) have not been defined, and dosing guidelines tailored to RDEB are lacking. Regular monitoring of serum zinc is advised in RDEB to guide supplementation, and current recommendations are largely extrapolated from general pediatric and adult practice [7,8]. Consistent with zinc’s classification as a Type 2 nutrient [32,48], circulating deficiency typically becomes evident once functional consequences are evident, supporting the need for clinically anchored preventive thresholds in RDEB.

The comparison with burn patients provides valuable clinical insight. Both conditions involve sustained metabolic demands, persistent inflammation, and significant exudative losses, all contributing to macro- and micronutrients depletion, including zinc [74,75]. In hospitalized burn patients, empirical high-dose zinc supplementation, often enteral or intravenous, is routinely recommended for 2–3 weeks, irrespective of baseline serum levels, improving wound healing and reducing infection rates and mortality [74,75,76]. While RDEB is chronic rather than acute, its metabolic profile (oxidative stress, immune dysregulation, and hypercatabolism) is strikingly similar. These parallels reinforce the rationale for a more proactive and sustained approach to zinc repletion in RDEB, aimed not only at restoring serum levels but also at counteracting ongoing losses and supporting systemic resilience.

The proposed threshold of 780 µg/L identified in this study achieved ≥80% sensitivity for major clinical complications, suggesting that it may be clinically more meaningful than the conventional cutoff for zinc deficiency. While this threshold could be considered conservative when compared with clinical practice in burn care, the chronic nature of RDEB requires sustained long-term management. Although OZS has a favorable safety profile, caution is warranted with prolonged high-dose use due to risks such as copper depletion [17,71,77]. In addition, concurrent iron supplementation may reduce the effectiveness of both micronutrients, given their competition for intestinal absorption [71,77]. Other common dietary components, including calcium, fiber, and phytates, often present in nutritional shakes prescribed to RDEB patients, can attenuate zinc absorption [71,77]. This reinforces the importance of nutritional expertise in guiding dose scheduling and timing of supplementation to maximize efficacy.

### 4.5. Limitations of This Study

The cross-sectional and single-center design of this study limits causal inference and generalizability. Residual confounding from variables that are difficult to quantify in this setting (such as dietary intake or socioeconomic factors) cannot be fully excluded. Prospective and interventional studies are therefore needed to confirm the value of the proposed preventive threshold (780 µg/L) and to determine the most effective zinc repletion strategies for improving clinical outcomes in RDEB.

## 5. Conclusions

This study provides the first disease-specific characterization of zinc status in RDEB, revealing strong associations with disease severity, anemia, impaired growth, chronic wounds, inflammation, and compromised immune competence. Zinc deficiency emerges as a clinically relevant and modifiable factor associated with complication burden, supporting the rationale for systematic monitoring and correction of deficiency as part of comprehensive supportive care and complication management in RDEB. As this study is cross-sectional, no causal or interventional conclusions can be drawn, and randomized controlled trials are required to determine whether zinc supplementation improves clinical outcomes and to define optimal repletion strategies.

## Figures and Tables

**Table 1:** Association of clinical outcomes with zinc deficiency. Predictive value of zinc deficiency, as a binary variable (deficient, no deficient) with cutoff 670 µg/L, for most common RDEB complications.

Clinical Outcomes	Deficient % (n/N)	NO Deficient % (n/N)	OR	*p*-Value	Sens %	Spec %	Youden’s J
**Cutaneous/Oral Mucosa Complications**							
BSA affected ≥25%	100 (29/29)	49(27/55)	∞	<0.0001	52	1	0.52
BSA affected ≥40%	82.7 (24/29)	23.6 (13/55)	15	<0.0001	65	89	0.54
Chronic wounds	79.3 (23/28)	31.5 (17/55)	10	<0.0001	57	86	0.44
Scarring alopecia	41.4 (12/29)	18.5 (10/54)	3.1	0.036	55	72	0.27
Oral mucosa blisters	82.8 (24/29)	75.9 (41/55)	1.6	NS	37	72	0.09
Premature tooth loss	65.5 (19/29)	21.8 (12/55)	6.8	0.0001	61	81	0.42
**Inflammatory and Infection Burden**							
CRP ≥ 15	100 (29/29)	41.8 (23/55)	∞	<0.0001	55	1	0.55
Leukocytosis	44.8 (13/29)	9 (5/55)	8.1	0.0004	72	76	0.48
Recurrent febrile episodes	44.8 (13/29)	18.5 (10/54)	3.6	0.02	57	73	0.3
Active cutaneous Infection	34.5 (10/29)	7.3 (4/55)	6.7	0.0038	79	74	0.53
History of Cutaneous infection	89.7 (26/29)	52.7 (29/55)	7.8	0.0007	47	90	0.37
HHI	39.3 (11/28)	14.5 (8/55)	3.8	0.025	58	72	0.3
**Psychological Complications**							
Anxiety	46.4 (13/28)	37 (20/55)	1.5	NS	39	68	0.07
Depression/Low mood	39.3 (11/28)	25.5 (14/55)	1.9	NS	44	69	0.13
**Anemia**	93.1 (27/29)	27.3 (15/55)	36	<0.0001	64	95	0.6

BSA = Body surface area; CRP = C-reactive protein; HHI = History of hospitalization due to serious infection, unresponsive to oral antibiotics or leading to sepsis. OR = Odds ratio; ∞ = OR not computable; all zinc-deficient patients had the outcome; *p*-value = Fischer test; Sens, Spec and Youden’s J = sensitivity, specificity and Youden’s J statistic (Sensitivi-ty + Specificity − 1) for zinc deficiency cutoff (670 µg/L).

## Data Availability

Data supporting this study are available to qualified academic researchers upon substantiated request to corresponding authors, provided the request aligns with the objectives specified in the participants’ consent agreements. Any data release will adhere to privacy protection protocols, such as deidentification, and will comply with applicable legal requirements.

## Supplementary Materials

The following supporting information can be downloaded at: https://www.mdpi.com/article/10.3390/nu18020232/s1. Supplementary Methods (Statistical analysis, Python-based analyses), Table S1 (Standard laboratory reference ranges), Supplementary Results (Oral Zinc Supplementation across age and severity; Figure S1), and Tables S2–S9 (expanded results and model details). Reference [78] are cited in the supplementary materials.

## Abbreviations

The following abbreviations are used in this manuscript:

AIC	Akaike Information Criterion
AUC	Area under the receiver operating characteristic curve
BSA	Body surface area
CRP	C-reactive protein
EBDASI	Epidermolysis bullosa disease activity and severity index
IL6	Interleukin-6
OR	Odds ratio
OZS	Oral zinc supplementation
RDEB	Recessive dystrophic epidermolysis bullosa
