# Supplementary material for: Zinc as a Biomarker of Nutritional Status and Clinical Burden in Recessive Dystrophic Epidermolysis Bullosa: Implications for Preventive Monitoring
Source: Nutrients. 2026 Jan 12;18(2):232. doi: 10.3390/nu18020232 (PMC12844941; doi:10.3390/nu18020232)
Supplement: Supplementary file 1 [file nutrients-18-00232-s001.zip › nutrients-4043350-supplementary.pdf]

Supplementary Material

Title: Zinc as a Biomarker of Nutritional Status and Clinical Burden in Recessive Dystrophic Epidermolysis Bullosa: Implications for Preventive Monitoring

Authors: Lucía Quintana-Castanedo, Rocío Maseda, Silvia Sánchez-Ramón, Nora Butta, Marta Molero-Luis, María G. Crespo, Antonio Buño, Sara Herráiz-Gil, Carlos León, Alberto Varas, Lidia M Fernández-Sevilla, Pilar Zuluaga, Raúl de Lucas, Marcela del Río, Ángeles Vicente, María J. Escámez, Rosa Sacedón

INDEX

1. Supplementary Methods

1.1. Statistical analysis.

1.2. Analysis performed using Python via the Data Analyst tool

1.3.1. Identification of zinc thresholds and diagnostic performance

1.3.2. Statistical Analysis of Zn and anemia risk

1.3.3. Statistical analysis of the factors contributing to hypozincaemia

1.3.4. Cross-Validation

1.3.5. Mediation analysis

1.3.6. Python Libraries

Table S1: Standard laboratory reference ranges

2. Supplementary Results

2.1 Oral Zinc supplementation (OZS) across age and severity groups

<u>**Figure and Tables**</u>

Figure S1. Oral Zinc supplementation (OZS) across age and severity groups

Table S2 Demographic characteristics of RDEB patients

Table S3. Zinc levels and status in patients distributed by Sex and RDEB severity (EBSADI score)

Table S4. Exploratory Identification of Zinc Thresholds Associated with 80% Sensitivity for Guiding Preventive Supplementation in RDEB.

Table S5. Diagnostic Performance of a 780 µg/L Serum Zinc Threshold for Guiding Preventive Supplementation in RDEB.

Table S6. Logistic regression models tested and excluded in the process of identifying the most accurate predictor combination for estimating anemia risk in RDEB

Table S7. Mediation Analysis of the Predictors of anemia risk CRP and BSA Through the Mediator Zinc

Table S8. Multiple linear regression (OLS) model tested and excluded in the process of identifying the most accurate predictors of blood Zinc levels.

Table S9. Mediation analysis of the predictors BSA and CRP through the mediator Albumin

1. Supplementary Methods

1.1. Statistical analysis.

Statistical analyses and visualization were conducted using a combination of conventional statistical software and AI-assisted tools. Data analysis and visualization were performed with Microsoft Excel 365, GraphPad Prism 8.0.2, and SPSS v25.0. Normality was assessed using the Kolmogorov–Smirnov–Lilliefors test (for $n > 30$) or the Shapiro–Wilk test (for $n < 30$). Associations between continuous variables were evaluated using Spearman's rank correlation coefficient (ρ). Comparisons of quantitative variables were made using the t-test when normality was confirmed, or the Mann–Whitney U test (two groups) and Kruskal–Wallis test (more than two groups), followed by Dunn's post hoc test, when distributions were non-normal. Categorical variables were compared using Pearson's chi-square test or Fisher's exact test as appropriate. Descriptive statistics included mean, standard deviation (SD), range, median, and interquartile range (IQR). P-values < 0.05 were considered statistically significant.

Identification and analysis of zinc thresholds, multivariate modeling, and mediation analyses were conducted using Python 3.11.4 within the Data Analyst environment (ChatGPT, OpenAI). All models were independently re-run and reviewed across sessions to ensure consistency, correct variable coding, and alignment with the study design. All procedures and results were critically reviewed and interpreted by the authors.

1.2. Analysis performed using Python via the Data Analyst tool

1.2.1. Identification of zinc thresholds and diagnostic performance

Serum zinc concentrations (µg/L) were tested against binary-coded major clinical outcomes. For each outcome, diagnostic parameters (sensitivity, specificity, predictive values (PPV), negative predictive value (NPV), and Youden's index (J = sensitivity + specificity − 1)) were calculated at every zinc value observed in the dataset. Thresholds were derived exclusively from real patient measurements, without interpolation.

Preventive candidate cutoffs were defined as the lowest zinc concentration that achieved ≥80% sensitivity, selecting the value with the highest Youden's index when multiple candidates were possible. After analysis, a fixed threshold of 780 µg/L was selected and evaluated across all outcomes to assess its diagnostic performance and clinical applicability.

1.2.2. Statistical Analysis of Zn and anemia risk

Logistic regression models were fitted to predict anemia risk using various combinations of predictors. Anemia status (binary: 1 = anemic, 0 = non-anemic) was modeled as the dependent variable. Candidate predictors included: serum zinc levels (µg/L), C-reactive protein (CRP, mg/L), percentage of BSA affected (%), transferrin saturation index (TSAT; binary: 1 =≤15, 0 =>15). Age (years), sex (binary, 1 =male, 0= female) we included as covariates. Univariate and multivariate models were fitted to assess the independent effects of these predictors. Interaction terms (Zinc×CRP and Zinc×TSAT) were tested to evaluate potential modifying effects but were excluded in

the final model due to non-significance. Coefficients (β), standard errors (SE), odds ratio (OR, e^{β}), 95% confidence intervals (CIs) for ORs and p-values, were reported. Model performance was evaluated using the Akaike Information Criterion (AIC), McFadden's R^2, and area under the ROC curve (AUC). Variance inflation factors (VIFs) were calculated to assess multicollinearity among predictors (values <5).

1.2.3. Statistical analysis of the factors contributing to hypozincaemia

Multiple linear regression (ordinary least squares, OLS) models were fitted to identify factors influencing serum zinc levels (µg/L). Predictors tested included: CRP (mg/L), BSA (%), IL6 (pg/mL), and serum albumin (g/dL). Age (years)and sex (binary, 1= male, 0= female) were included as covariates. After an exploratory analysis, the interaction term CRPxBSA was calculated by multiplying the two variables and added to the final model. For each predictor, coefficients (β), SE, T-statistic (t, β/SE); p- (H_0: $\beta = 0$); 95% CIs and VIFs of each predictor were reported. Models were compared using adjusted R^2 and AIC, using the same patient set (excluding those without IL6 measurements).

1.2.4. Cross-Validation

All models were validated using 5-fold cross-validation. For each fold, model performance was evaluated on a test subset comprising 20% of the data. Metrics reported included the mean, standard deviation (SD), and range across folds for AUC, classification accuracy, sensitivity, and specificity. OLS models were validated using mean R^2 and mean squared error (MSE).

1.2.5. Mediation analysis

Mediation analyses were performed to examine whether the associations between clinical predictors and anemia risk (or zinc levels) were mediated by intermediate biomarkers. Specifically, we tested whether zinc mediated the relationship between inflammation (CRP) or extent of skin involvement (%BSA) and the presence of anemia, and whether albumin mediated the relationship between CRP or %BSA and circulating zinc levels.

The mediation framework followed the steps described by Baron and Kenny, using a series of OLS or logistic regression models depending on the outcome. In each analysis, three models were constructed: (1) the outcome regressed on the predictor alone; (2) the mediator regressed on the predictor; and (3) the outcome regressed on both the predictor and the mediator. All models were adjusted for age and sex. For analyses where the outcome was binary (anemia status), logistic regression was used in steps 1 and 3.

To test the significance of the indirect effect (product of paths a and b), the Sobel test was applied, using the formula: $Z = (a \cdot b) / \sqrt{(b^2 \cdot sa^2 + a^2 \cdot sb^2)}$; where *a* and *b* are the unstandardized regression coefficients for the predictor → mediator and mediator → outcome paths, and *sa* and *sb* are their respective standard errors.

1.2.6. Python Libraries

Libraries used included: pandas for data processing (v2.0.3), NumPy for numerical operations, statsmodels (v0.14.0) for regression modeling and mediation analysis, scikit-learn *(*StratifiedKFold for anemia prediction or KFold for zinc levels prediction*)* for

cross-validation and performance metrics, scipy (v1.11.1) for statistical tests, and python-docx for table generation.

Table S1: Standard laboratory reference ranges

**Parameter**	**units**	**NR**		
**Zinc**	**µg/L**	670-1200		
**CRP**	mg/L	<5		
**Iron**	**µg/dL**	M=<12y=40-120; ≥12y:65-175<br>F=<12y=40-120; ≥12y:50-170		
**Leukocytes**	**COUNT x 10^3cel/µL**	1y-12y : 4.8-15<br>12y-18y: 4.2-11.4<br>18y-65y: 3.9-10.2<br>>65y: 3.6-10.5		
**TSAT**	**%**	>15		
**Albumin**	**g/dL**	1y-14 y: 3.8 - 5.4<br>>14y: 2.9 - 5.2		
**Hemoglobin levels to diagnose anemia (g/dL)[a]**				
**Age/sex**	**Non-anemia**	**Anemia**		
		**Mild**	**Moderate**	**severe**
1y	≥10.5	9.5-10.4	7.0-9.4	<7.0
2y-<5y	≥11.0	10.0-10.9	7.0-9.9	<7.0
5y-11y	≥12.0	11.0-11.9	8.0-10.9	<8.0
12-14y	≥12.0	11.0-11.9	8.0-10.9	<8.0
>14y Women	≥12.0	11.0-11.9	8.0-10.9	<8.0
>14y Men	≥13.0	11.0-12.9	8.0-10.9	<8.0

NR= Normal range according to laboratory values; M= male; F=female
TSAT= Transferrin saturation index; y= years old
[a] Adapted from *"Guideline on haemoglobin cutoffs to define anaemia in individuals and populations*". Geneva: World Health Organization; 2024. Licence: CC BY-NC-SA 3.0 IGO". https://www.who.int/publications/i/item/9789240088542

Supplementary Results

Oral Zinc supplementation (OZS) across age and severity groups

Only a minority of patients across all age groups reported receiving OZS (18/84, 17 severe, 1 moderate) (Figure S1). OZS did not consistently correlate with the patient's Zn status or requirements according to their % of BSA affected, severity or inflammatory status and many patients with BSA affected >25% or CRP levels above 15 mg/L were not on OZS. Notably, a substantial proportion of Zn-deficient patients were not receiving OZS: 58% of children and adolescents and 62.5% of adults. In the young children group (<5 y), despite most having zinc near the lower limit of the NR (Fig), only one patient (7.7%) was on OZS. In contrast, 27% of the children and adolescents (5-<18 y) and 20.6% of the adults reported taking zinc supplements. Among the patients receiving supplementation, many remained Zn deficient: 50% of children and adolescents, and 85.7% of adults.

a

ZINC STATUS	TOTAL	SEX		RDEB SEVERITY-EBDASI CATEGORIES		
		M	F	Mild	Moderate	Severe
Deficiency	11/29 (38%)	4/17 (24%)	7/12 (58%)	-	-	11/29 (38%)
NR	7/54 (13%)	3/26 (12%)	4/28 (14%)	0/13 (0%)	1/22 (4.5%)	6/19 (37%)
Excess	0/1 (0%)	0/1 (0%)	-	-	-	0/1 (0%)
∑ TOTAL	18/84 (21%)	7/44 (16%)	11/40 (28%)	0/13(0 %)	1/22 (4.5%)	17/49 (35%)

b

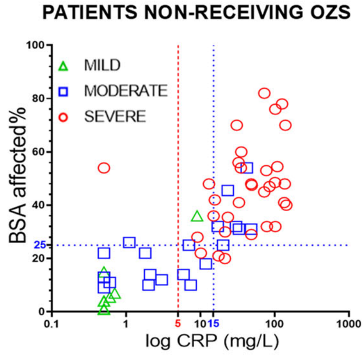

c **d**

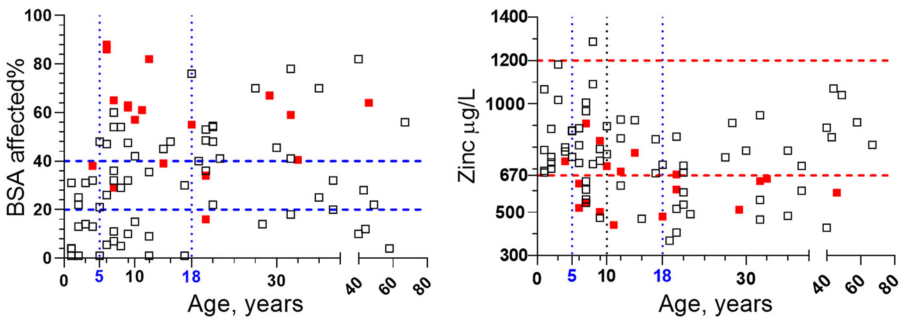

Figure S1.- Oral zinc supplementation (OZS) in the RDEB cohort

(a) Patients taking OZS (n, %) by zinc status (deficiency, normal range (NR), and excess), sex and EBDASI severity categories.

(b) Clinical characteristics, body surface area (BSA) affected (%), C- reactive protein (CRP, mg/L), and EBASI severity category of patients non-receiving OZS

(c-d) BSA affected and Zinc levels across age (years) of patients receiving (red squares) or not (open squares) OZS.

Table S2.- Demographic characteristics of RDEB patients

		**Total,** n (%)	**F** n (%)	**Age** Mean±SD (Range)	**AGE GROUPS**		
					**Young children (1-<5 y)** n (%)	**Children & Adolescents (5-<18 y)** n (%)	**Adults (≥18)** n (%)
**Total**		84 (100)	40/84 (48)	17.1±14 (1-67)	13/84 (15)	37/84 (44)	34/84 (40)
**EBDASI SEVERITY CATEGORIES**	**Mild**	13/84 (16)	6/40 (15)	11.4±15.3 (1-58)	4/13 (31)	7/37 (19)	2/34 (6)
	**Moderate**	22/84 (26)	10/40 (25)	16.5±15.8 (1-49)	8/13 (62)	5/37 (14)	9/34 (26)
	**Severe**	49/84 (58)	24/40 (60)	18.8±13.6 (4-67)	1/13 (8)	25/37 (68)	23/34 (68)

EBDASI (Epidermolysis Bullosa Disease Activity and Scarring Index) score: The involvement of the skin, scalp, mucous membranes, nails, and other epithelialized surfaces are sub-scored in terms of activity (0-276) and damage (0-230 points). According to the total EBDASI, calculated by adding these sub-scores, the patients were stratified into 3 categories: mild (0-42 points), moderate (43-106) and severe (107-506). (Loh et al. J Am Acad Dermatol 2014 Jan;70(1):89-97.e1-13.; DOI: 10.1016/j.jaad.2013.09.041)

Note: Percentages in this table are rounded to whole numbers, hence totals may slightly exceed or fall below 100% due to rounding.

Table S3.- Zinc levels and status in patients distributed by Sex and RDEB severity (EBSADI score).

		**SEX**		**RDEB SEVERITY-EBDASI CATEGORIES**		
	**Total** (n= 84)	**M** (n=44)	**F** (n=40)	**Mild** (n=13)	**Moderate** (n=22)	**Severe** (n=49)
**ZINC LEVELS (µg/L)**						
Mean ± SD (Range)	735±188 (369-1287)	730±206 (369-1287)	740±169 (429-1091))	892±110 (683-1091)	850±142 (674-1181)	642±167 (369-1287)
**ZINC STATUS (NR=670-1200 µg/L) n (%)**						
**Deficiency**	29/84 (35)	17 (39)	12/40 (30)	0 (0)	0 (0)	29 (59)
**NR**	54/84 (64)	26 (59)	28 (70)	13/13 (100)	22/22 (100)	19 (39)
**Excess**	1/84 (1,2)	1 (2)	0 (0)	0 (0)	0 (0)	1 (2)

EBDASI (Epidermolysis Bullosa Disease Activity and Scarring Index) **categories**: **Mild**= ≤42 points, M**oderate**= 43-106 points, **Severe**= ≥107 points).

(

Table S4. Exploratory Identification of Zinc Thresholds Associated with 80% Sensitivity for Guiding Preventive Supplementation in RDEB.

CLINICAL OUTCOME	Age Group	Zinc Threshold (μg/L)	Sens (%)	Spec (%)	PPV (%)	NPV (%)	Youden's J
**CUTANEOUS / ORAL MUCOSA COMPLICATIONS**							
**BSA affected ≥ 25%**	Total	**784**	80	79	88	67	0.59
	Children	**787**	81	84	89	73	0.65
	Adults	**784**	80	67	87	55	0.47
**BSA affected ≥ 40%**	Total	**755**	81	66	65	82	0.47
	Children	**710**	80	78	63	89	0.58
	Adults	**714**	80	79	84	73	0.59
**Chronic wounds**	Total	**846**	80	35	53	65	0.15
	Children	**909**	80	20	40	60	0.0
	Adults	**784**	80	54	73	64	0.34
**Scarring alopecia**	Total	**787**	82	44	35	87	0.26
	Children	**768**	80	58	32	92	0.38
**Premature tooth loss**	Total	**768**	81	57	52	83	0.37
	Children	**755**	82	64	39	93	0.46
**INFLAMMATORY AND INFECTION BURDEN**							
**CRP ≥ 15**	Total	**755**	79	87	91	71	0.66
	Children	**780**	79	77	82	74	0.56
	Adults	**715**	80	100	100	64	0.8
**Leukocytosis**	Total	**897**	80	16	77	19	-0.04
	Children	**897**	79	28	87	18	0.08
	Adults	**912**	82	8	62	20	-0.1
**Recurrent febrile episodes**	Total	**755**	78	55	40	87	0.33
	Adults	**784**	80	39	36	82	0.19
**Active cutaneous infection**	Total	**642**	79	79	42	95	0.57
**History of cutaneous infection**	Total	**792**	80	66	82	63	0.46
	Children	**799**	81	63	70	75	0.43
**HIH**	Total	**714**	79	66	41	92	0.45
	Children	**691**	82	85	60	94	0.66
**Anemia**	Total	**722**	81	86	85	82	0.67
	Children	**738**	82	86	82	86	0.68
	Adults	**683**	80	86	89	75	0.66

BSA= Body surface area; **CRP=** C-reactive protein; **HHI** History of hospitalization due to serious infection, unresponsive to oral antibiotics or leading to sepsis
Sens = Sensitivity**; Spec =** Specificity**; PPV =** Positive Predictive Value**; NPV =** Negative Predictive Value**; Youden's J =** Youden's Index (Sensitivity + Specificity − 1**)**

Thresholds are reported only when a zinc level achieving approximately 80% sensitivity (±2%) could be identified. Missing values indicate that no threshold met this criterion, typically due to insufficient sample size, low event count, or weak discriminatory performance within the specified subgroup

Table S5. Diagnostic Performance of a 780 µg/L Serum Zinc Threshold for Guiding Preventive Supplementation in RDEB.

CLINICAL OUTCOME	Group	Sens (%)	Spec (%)	PPV (%)	NPV (%)	Youden's J
**CUTANEOUS / ORAL MUCOSA COMPLICATIONS**						
**BSA affected ≥ 25%**	Total	79	79	88	65	0.57
	Children	77	84	89	70	0.62
	Adults	80	67	87	54	0.47
**BSA affected ≥ 40%**	Total	86	62	64	85	0.48
	Children	82	61	52	87	0.43
	Adults	90	64	78	82	0.54
**Chronic wounds**	Total	70	51	57	65	0.21
	Children	60	50	44	65	0.1
	Adults	80	54	73	64	0.34
**Scarring alopecia**	Total	77	48	35	85	0.25
	Children	80	52	30	91	0.33
	Adults	75	38	41	73	0.13
**Premature tooth loss**	Total	81	53	50	82	0.33
	Children	91	56	37	96	0.47
	Adults	75	43	65	54	0.18
**INFLAMMATORY AND INFECTION BURDEN**						
**CRP ≥ 15**	Total	83	81	88	74	0.64
	Children	79	77	82	74	0.56
	Adults	88	89	96	73	0.77
**Leukocytosis**	Total	54	21	70	12	-0.25
	Children	51	29	82	9	-0.2
	Adults	59	17	56	18	-0.24
**Recurrent febrile episodes**	Total	83	50	39	88	0.33
	Children	85	57	41	91	0.41
	Adults	80	39	36	82	0.19
**Active cutaneous Infection**	Total	93	47	26	97	0.4
	Children	100	52	22	100	0.52
	Adults	88	38	30	91	0.26
**History of Cutaneous infection**	Total	74	69	82	59	0.44
	Children	73	67	70	70	0.4
	Adults	76	80	96	36	0.56
**HHI**	Total	95	51	36	97	0.46
	Children	100	59	41	100	0.59
	Adults	88	38	30	91	0.26
Anemia	Total	93	74	78	91	0.67
	Children	86	71	70	87	0.58
	Adults	100	79	87	100	0.79

BSA= Body surface area**; CRP=** C-reactive protein; **ACI**= Active cutaneous infection; **HCI**= History of cutaneous infection; **HHI**= History of hospitalization because of serious infection, nonresponsive to oral antibiotics or sepsis
Sens = Sensitivity**; Spec =** Specificity**; PPV =** Positive Predictive Value**; NPV =** Negative Predictive Value**; Youden's J =** Youden's Index (Sensitivity + Specificity − 1**)**

Table S6.- Logistic regression models tested and excluded in the process of identifying the most accurate predictor combination for estimating anemia risk in RDEB

Predictor	β	SE	OR	95% CI (OR)	p-value
**Model 1: Zinc Only**					
Intercept	11.88	2.66			
Zinc	-0.0163	0.0036	0.98	[-0.02, -0.01]	<0.0001
**Model 2: All candidate predictors**					
Intercept	9.38	5.8063			
Zinc	-0.017	0.007	0.98	[0.97, 1.0]	0.01
CRP	0.033	0.018	1.03	[1.0, 1.07]	0.065
BSA affected	-0.0035	0.036	1.0	[0.93, 1.07]	0.92
TSAT	3.89	1.68	48.91	[1.82, 1312.91]	0.02
age	-0.065	0.05	0.94	[0.86, 1.03]	0.16
sex	-0.55	1.06	0.58	[0.07, 4.57]	0.60
**Model 3: Without CRP**					
Intercept	8.99	4.59			
Zinc	-0.018	0.006	0.98	[0.97, 0.99]	0.002
BSA affected	0.040	0.03	1.04	[0.99, 1.09]	0.12
TSAT	4.22	1.64	68.03	[2.77, 1685.9]	0.01
age	-0.058	0.04	0.94	[0.88, 1.02]	0.12
sex	-0.48	0.94	0.62	[0.1, 3.9]	0.61
**Model 5: Without TSAT**					
Intercept	10.2390	4.34			
Zinc	-0.0144	0.005	0.99	[0.98, 1.0]	0.004
CRP	0.043	0.02	1.04	[1.0, 1.08]	0.03
BSA affected	0.0026	0.03	1.0	[0.94, 1.07]	0.94
age	-0.0626	0.04	0.94	[0.87, 1.02]	0.12
Sex	-1.29	0.89	0.28	[0.05, 1.58]	0.15
**Model 6: Without CRP or BSA**					
Intercept	12.25	4.12			
Zinc	-0.02	0.006	0.98	[0.97, 0.99]	0.0003
TSAT	4.24	1.46	69.41	[3.94, 1224.15]	0.004
age	-0.05	0.036	0.95	[0.89, 1.02]	0.17
Sex	-0.40	0.89	0.67	[0.12, 3.86]	0.65
**Model 7: Without Zinc**					
Intercept	-5.69	1.92			
CRP	0.05	0.02	1.05	[1.01, 1.08]	0.005
BSA affected	0.04	0.03	1.04	[0.98, 1.11]	0.2
TSAT	3.7	1.47	40.45	[2.25, 727.78]	0.012
age	-0.04	0.03	0.96	[0.91, 1.02]	0.21
sex	-0.01	0.8	0.99	[0.2, 4.76]	0.99

Candidate predictors: Zinc (μg/L), C-Reactive protein (CRP, mg/L), Body surface area affected (BSA, %), transferrin saturation index (binary, coded ≤ 15%=1, >15%=0), age (years), sex (binary, coded male=1, female=0)

OR= odds ratio (e^{β}) The OR represents the change in odds of anemia per unit increase of the predictor variable (for continuous variables) or relative to the reference category (for categorical variables). Values >1 indicate increased odds of anemia, and values <1 indicate reduced odds.

p-value= statistical significance of each predictor

95% CI (OR): confidence interval for OR estimates, reported as [lower, upper].

Table S7. Mediation Analysis of the Predictors of anemia risk CRP and BSA Through the Mediator Zinc

	CRP → Zinc → Anemia	BSA → Zinc → Anemia
**a (Predictor → Zinc)**	-2.12	-4.94
**b (Zinc → Anemia)**	-0.0163	-0.0163
**a × b (Indirect Effect)**	0.03	0.08
**Sobel Test Statistic (Z)**	3.75	3.76
**P-value (Sobel)**	0.00018	0.00017

Table S8.- Multiple linear regression (OLS) model tested and excluded in the process of identifying the most accurate predictors of blood Zinc levels.

Predictor	β	SE	t	p-value	CI 95	VIF
**Model 1: All candidate predictors**						
**Intercept**	85.09	191.9	0.44	0.66	[-297.5,467.6]	
**BSA %**	-1.48	1.15	-1.29	0.20	[-3.77, 0.81]	3.69
**CRP mg/L**	0.26	0.49	0.57	0.57	[-0.64, 1.15]	3.1
**IL6 pg/mL**	-0.001	0.008	-0.18	0.88	[-0.017, 0.015]	1.02
**Alb g/dL**	179.15	40.14	4.46	**<0.000**	[99.14, 259.2]	3.9
**Age, years**	-0.73	0.98	-0.75	0.46	[-2.68, 1.2]1	1.1
**Sex, M/F**	-46.77	27.86	-1.68	0.098	[-102.3, 8.77]	1.03
**Model 2: Whitout Albumin**						
**Intercept**	928.28	37.84	24.53	0.000	[852.9, 1003.7]	
**BSA affected %**	-4.29	1.08	-3.98	**<0.000**	[-6.44, -2.14]	2.57
**CRP mg/L**	-0.61	0.45	-1.35	0.18	[-1.52, 0.29]	2.5
**IL6 pg/mL**	-0.003	0.009	-0.28	0.78	[-0.02, 0.015]	1.02
**Age, years**	0.37	1.06	0.35	0.73	[-1.75, 2.48]	1.03
**Sex, M/F**	-44.01	31.26	-1.41	0.16	[-106.3, 18.3]	1.03
**Model 2: Whitout IL6**						
**Intercept**	36.99	190.62	0.19	0.84	[-342.9, 416.89]	
**BSA affected %**	-1.49	1.14	-1.30	0.20	[-3.76, 0.79]	3.68
**CRP mg/L**	0.26	0.44	0.59	0.56	[-0.62, 1.14]	3.08
**Alb g/dL**	179.38	39.84	4.50	**<0.000**	[99.98, 258.78]	3.89
**Age, years**	-0.73	0.97	-0.75	0.46	[-2.66, 1.20]	1.10
**Sex, M/F**	-46.77	27.54	1.68	0.097	[-8.53,1 01.25]	1.02

Candidate predictors for Zinc levels (μg/L), C-Reactive protein (CRP, mg/L), Body surface area affected (BSA, %), Albumin (g/dL), IL6 (pg/mL), age (years), sex (binary, coded male =0, Female =1)

β= coefficients unstandardized, representing the change in zinc concentration (μg/L) per unit change in the predictor variable.

SE= coefficient's standard error; **t=** T-statistic (β/SE); **P-val**= P- value for testing β ≠ 0; **95% CI=** confidence interval; **VIF=** Variance Inflation Factor

Table S9. Mediation analysis of the predictors BSA/ CRP through the mediator Albumin

BSA → Albumin → Zinc		CRP → Albumin → Zinc		CRP × BSA → Albumin	
a (BSA → Albumin)	-0.024	a (CRP → Albumin)	-0.010	a (CRP×BSA → Albumin)	-0.000
b (Albumin → Zinc)	163.88	b (Albumin → Zinc)	197.49	b (Albumin → Zinc)	254.42
a × b (Indirect Effect)	-3.92	a × b (Indirect Effect)	-1.95	a × b (Indirect Effect)	-0.037
Sobel Test Statistic	-4.40	Sobel Test Statistic	-5.247	Sobel Test Statistic	-6.36
P-value (Sobel)	0.000	P-value (Sobel)	0.000	P-value (Sobel)	0.000
